# Flow Rates Measurement and Uncertainty Analysis in Multiple-Zone Water-Injection Wells from Fluid Temperature Profiles

**DOI:** 10.3390/s16071077

**Published:** 2016-07-13

**Authors:** José E. O. Reges, A. O. Salazar, Carla W. S. P. Maitelli, Lucas G. Carvalho, Ursula J. B. Britto

**Affiliations:** 1 Laboratório de Avaliação e Medição em Petróleo, Departamento de Engenharia de Computação e Automação, Universidade Federal do Rio Grande do Norte, Natal 59072970, Brazil; 2 Laboratório de Automação em Petróleo, Departamento de Engenharia de Petróleo, Universidade Federal do Rio Grande do Norte, Natal 59072970, Brazil; carlamaitelli@gmail.com (C.W.S.P.M.); lucas.gdcarvalho@bct.ect.ufrn.br (L.G.C.); ursulabritto@gmail.com (U.J.B.B.)

**Keywords:** flow sensors, oil and gas industry, multiple-zone water-injection wells, wellbore heat transmission, measurement uncertainty

## Abstract

This work is a contribution to the development of flow sensors in the oil and gas industry. It presents a methodology to measure the flow rates into multiple-zone water-injection wells from fluid temperature profiles and estimate the measurement uncertainty. First, a method to iteratively calculate the zonal flow rates using the Ramey (exponential) model was described. Next, this model was linearized to perform an uncertainty analysis. Then, a computer program to calculate the injected flow rates from experimental temperature profiles was developed. In the experimental part, a fluid temperature profile from a dual-zone water-injection well located in the Northeast Brazilian region was collected. Thus, calculated and measured flow rates were compared. The results proved that linearization error is negligible for practical purposes and the relative uncertainty increases as the flow rate decreases. The calculated values from both the Ramey and linear models were very close to the measured flow rates, presenting a difference of only 4.58 m³/d and 2.38 m³/d, respectively. Finally, the measurement uncertainties from the Ramey and linear models were equal to 1.22% and 1.40% (for injection zone 1); 10.47% and 9.88% (for injection zone 2). Therefore, the methodology was successfully validated and all objectives of this work were achieved.

## 1. Introduction

**Figure 1:**
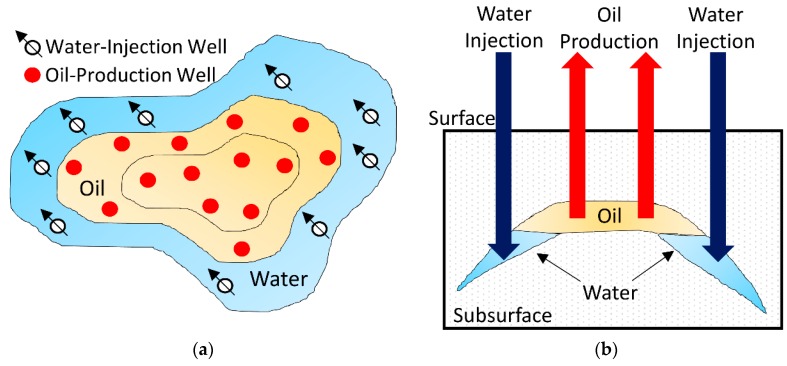
A common scheme for injection of water in oil fields. (**a**) Top view of an oil field composed of water-injection and oil-production wells; (**b**) The water flooding process. The injected water moves the oil up, increasing the oil production.

The oil reservoirs whose mechanisms are inefficient and retain large quantities of hydrocarbons after exhaustion of their natural energy are strong candidates for employment of processes aiming to achieve further recovery. In conventional methods, the oil recovery factor is enhanced by increasing the reservoir pressure. A fluid is injected inside the reservoir in order to move the oil out of the rock pores. Among the conventional oil recovery methods is the injection of water [1]. Figure 1 shows a common scheme for the injection of water in oil fields. The top view of an oil field composed of water-injection and oil-production wells is presented in Figure 1a. The water flooding process is illustrated in Figure 1b. The injected water moves the oil up, increasing the oil production.

**Figure 2:**
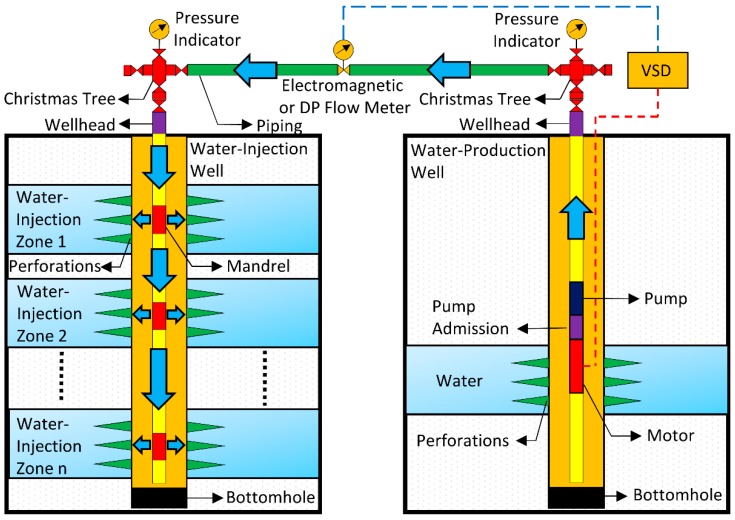
A typical water-injection system composed of a multiple-zone water-injection well and a water-production well. The total flow of water is measured and controlled on the surface. The injected flow rates are controlled by mechanical downhole flow regulators and there is no real-time, in-line flow rates measurement in the injection zones. VSD refers to a variable speed drive. DP refers to differential pressure.

The water for injection may be obtained from: subsurface, through wells drilled for this purpose; rivers; lakes; the sea; or produced water, a byproduct of oil and gas exploration and production [1]. The water-injection well may have one or more injection zones. The multiple-zone injection technique is desirable to reduce investment costs, since it uses a single well for simultaneous injection in various areas. However, this technique presents operational problems in relation to the monitoring of individual water-injection flow rates. A typical water-injection system composed of a multiple-zone water-injection well and a water-production well is shown in Figure 2.

The total flow of water is easily measured and controlled on the surface. In common approaches, the measurement of the total flow is performed by electromagnetic or differential pressure (DP) meters. Moreover, a variable speed drive (VSD) motor may be used to control the flow through a submersible centrifugal pump installed in the water-production well. On the other hand, it is not simple to control and measure the injected flow rates at subsurface in the multiple-zone water-injection well. Normally, the injected flow rates are controlled by mechanical downhole flow regulators into mandrels installed at each injection zone. However, this approach presents some issues. The mechanical parts have a low reliability. Therefore, a simple mechanical failure may compromise the injection quotas specified by the reservoir engineering team.

This problem is compounded by the fact that, in most cases, there is no real-time, in-line flow rates measurement in the injection zones. Periodically, a flow sensor is inserted within the injection column to measure the downhole flow rates. Next, the collected data are made available for analysis. This process is costly and does not offer information in sufficient frequency for a good monitoring of the water-injector wells. Therefore, faults in the mechanical regulators may stay undetected for a long time, drastically reducing the efficiency of the recovery method.

Meanwhile, several techniques to measure the temperature profile along the wellbore have been used. One of these techniques is the optic fiber Distributed Temperature Sensing (DTS). In this approach, an optic fiber is permanently inserted within the injection column, making possible the in-line acquisition of the fluid temperature profile along the water-injection well at shorter intervals of time, or even in real-time. In this context, the following questions arise: is it possible to estimate the flow rates in each injection zone from the fluid temperature analysis along the water-injection well? Furthermore, if it is possible, what is the measurement uncertainty?

The main objective of this work is to present a methodology to measure the flow rates into multiple-zone water-injection wells from fluid temperature profiles and estimate the measurement uncertainty. In this sense, the specific objectives are: to describe the fluid temperature along wellbore as a function of the injected flow rates; to obtain a closed-form solution for the injected flow rates as a function of the fluid temperature profile; to perform an uncertainty analysis to understand how errors in the measured temperatures influence the calculated flow rates; to develop a computer program to calculate the injected flow rates; and to validate the methodology from experimental temperature profiles obtained in the field.

## 2. Literature Review

The concepts, techniques and applications of downhole temperature measurements have been discussed in several papers since the 1950s. Qualitative surveys [2] have been applied in the interpretation of temperature logs through wellbores providing useful information as to the location of water-intake strata [3] and the direction and amount of fluid that is moving in a bore hole [4].

The first quantitative analysis of wellbore heat transmission was performed by Ramey (1962). He developed a mathematical model for incompressible liquid and ideal gas, describing the fluid temperature along a wellbore assuming that heat transfer is steady-state. The solution may be applied to a large variety of wellbore heat problems involving different types of well completions and operating methods [5]. Alves et al. (1992) proposed a unified model for predicting flowing temperature distribution in wellbores and pipelines. This model can be applied to pipelines or production and injection wells, under single or two-phase flow, over the entire inclination angle range from horizontal to vertical, with compositional and black-oil fluid models [6]. Maubeuge et al. (1994) described a model that takes into account the temperature effects due to the decompression of the fluid and the frictional heating that occurs in the formation. Moreover, the model predicts flow rates and temperatures in the wellbore and formation in response to the input pressure history [7].

Hasan et al. (2007) presented an analytic model for computing the fluid temperature in complex wells [8]. Based on this work, Izgec et al. (2010) proposed two methods for flow rate estimation from wellhead pressure and temperature data. The first method involves the entire wellbore in that both pressure-drop and the attendant heat-transfer calculations are done. In the second method, a single point in the wellbore temperature profile is used to estimate the flow rates [9].

From the last decade, optic fiber Distributed Temperature Sensing (DTS) technology has been widely used for monitoring injection profile in wellbores [10,11,12]. Muradov and Davies (2012) proposed a workflow for zonal flow rate and pressure allocation in wells equipped with DTS. The workflow is based on the assumption that the depths of the production intervals are known and that the only measurements taken are the distributed temperatures along the wellbore. The method was demonstrated on synthetic, production well data [2].

## 3. Materials and Methods

### 3.1. Theoretical Development

Initially, the classical model proposed by Ramey (1962) was examined to perform the zonal flow rates measurement from fluid temperatures. This model describes the fluid temperature along the wellbore as a transcendental and exponential function of flow. Therefore, the zonal flow rates may be iteratively calculated from this function. However, it makes an uncertainty analysis difficult.

For this reason, the Ramey equation was expanded in Taylor series and linearized. Then, a closed-form solution for the zonal flow rates as functions of fluid temperature was obtained to estimate the measurement uncertainty. In this approach, the systematic error, inherent to the linearization of the Ramey model, was not taken in account. The random error was estimated from the propagation of uncertainties for the measured temperatures at each depth along the wellbore.

#### 3.1.1. The Ramey Model for Fluid Temperature along Water-Injection Wells

**Figure 3:**
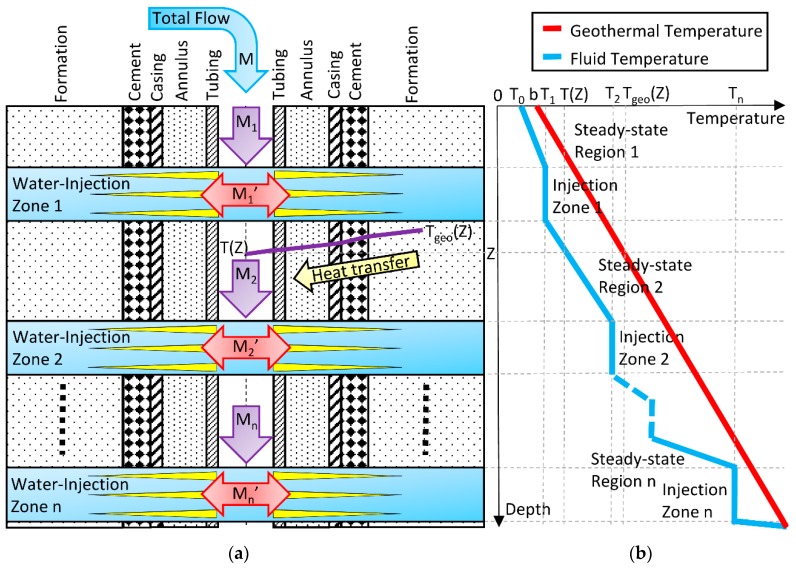
Flow and temperature along a vertical multiple-zone water-injection well. (**a**) Longitudinal section of the well. At center of wellbore, a fluid (water) flows down along the tubing. Due to the difference between fluid and geothermal temperatures, heat transmission occurs from the formation (surround rock) to the fluid across the cement, casing, annulus, and tubing; (**b**) The plot illustrates the qualitative behavior of the fluid and geothermal temperatures along the wellbore.

Consider the flow along a vertical multiple-zone water-injection well whose longitudinal section is illustrated in Figure 3. The total flow rate is *M*. The flow rates in the steady-state regions are *M*_1_, *M*_2_, …, *M*_n_. The injected flow rates are *M*_1_′, *M*_2_′, …, *M*_n_′. The fluid and geothermal temperatures at surface are T_0_ and b, respectively. Due to the difference between the fluid and geothermal temperatures along the wellbore, *T*(*Z*) and *T*_geo_(*Z*), as water flows down, heat transmission occurs from the formation to the fluid across the cement, casing, annulus, and tubing. Therefore, the fluid temperature increases along the wellbore. Into each injection zone the fluid temperature is considered approximately constant and equal to *T*_1_, *T*_2_, …, *T*_n_. Moreover, as part of the total flow is injected into the upper zones, the flow rate decreases along the wellbore (*M*_1_ > *M*_2_ > … > *M*_n_) and the fluid temperature gradient increases. After the last water-injection zone the flow rate is zero and the fluid temperature follows the geothermal temperature. The Ramey model [5] for fluid temperature as a function of depth (Z) in each steady-state region is given by:

(1) $$T\left(Z\right)=aZ+b_{i}-aA_{i}+\left({T_{0}}_{i}+aA_{i}-b_{i}\right)e^{-\frac{Z}{A_{i}}},\;i=1,\;2,\;\ldots ,\;n$$

where n is the number of injection zones, a is the geothermal gradient, $b_{i}$ is the initial geothermal temperature, $A_{i}$ is the relaxation distance, and ${T_{0}}_{i}$ is the initial fluid temperature in each steady-state region i. In turn, the relaxation distance $A_{i}$ [13] is equal to:

(2) $$A_{i}=M_{i}\times \left(\frac{c_{p}}{2\pi r_{tub}U_{c}}\right),\;i=1,\;2,\;\ldots ,\;n$$

where $M_{i}$ is the fluid injection mass flow rate in each steady-state region i, $c_{p}$ is the specific heat at constant pressure of fluid, $r_{tub}$ is the inside radius of tubing, and $U_{c}$ is the over-all heat-transfer coefficient described in Appendix A.

A qualitative analysis of Equations (1) and (2) indicates that the mass flow rate $M_{i}$ is proportional to the relaxation distance $A_{i}$. However, the Ramey model for fluid temperature is a transcendental, exponential function of $A_{i}$. Consequently, once the fluid temperature profile T(Z) is known, the calculation of the mass flow rate $M_{i}$ by Equation (1) has to be iteratively performed.

Moreover, as discussed in Appendix A, the term in parentheses in Equation (2) depends on thermal properties of fluid and earth; injection time; and constructive characteristics of the well (dimensions and materials of tubing, casing, and cement). Oftentimes, some of these characteristics are not easily known. Therefore, to calculate the mass flow rate $M_{i}$ it is necessary to suppose them.

#### 3.1.2. Estimating the Flow Rates from a Fluid Temperature Profile Using the Ramey Model

A method to estimate the flow rates in a multiple-zone water-injection well using the Ramey model is described below:

1. Identify the injection zones and determine the steady-state regions from the temperature profile;
2. Determine the total flow at surface, the geothermal temperature at surface, and the time of injection;
3. Determine density, viscosity, specific heat, and thermal conductivity of fluid;
4. Determine diameters and thermal conductivities of tubing, casing, and cement;
5. Determine thermal diffusivity, outside radius, and thermal conductivity of formation;
6. Calculate, iteratively from (1) and (2), the flow rates *M*_1_, *M*_2_, …, *M*_n_ in each steady-state region;
7. Calculate the flow rates *M*_1_′, *M*_2_′, …, *M*_n_′ in each injection zone:
  (3) $${M_{i}}^{\prime }=M_{i}-M_{i+1},\;i=1,\;2,\;\ldots ,\;n$$

#### 3.1.3. A Linear Model for Fluid Temperature along Water-Injection Wells

As was discussed in Section 3.1.1, because the fluid temperature is a transcendental, exponential function of the relaxation distance $A_{i}$, the mass flow rate $M_{i}$ calculation has to be iteratively performed. It makes the flow rates calculations difficult and complicates the analysis of the measurement uncertainty. Therefore, it would be useful to obtain a closed-form solution for the injected flow rates as a function of the fluid temperature profile.

Expanding the Equation (1) in Taylor series (at $Z=Z_{i}$) and rearranging the resultant equation to consider a linear approximation, as described in Appendix B, it follows that fluid temperature as a depth function is:

(4) $$T\left(Z\right)\approx T\left(Z_{i}\right)+\left[\frac{T_{geo}\left(Z_{i}\right)-T\left(Z_{i}\right)}{A_{i}}\right]\left(Z-Z_{i}\right),\;i=1,\;2,\;\ldots ,\;n$$

where $T\left(Z_{i}\right)$ is the fluid temperature at $Z_{i}$; $T_{geo}\left(Z_{i}\right)$ is the geothermal temperature at $Z_{i}$; and the relaxation distance $A_{i}$ is given by Equation (2). Solving Equation (4) for $A_{i}$:

(5) $$A_{i}=\left[\frac{T_{geo}\left(Z_{i}\right)-T\left(Z_{i}\right)}{T\left(Z\right)-T\left(Z_{i}\right)}\right]\times \left(Z-Z_{i}\right),\;i=1,\;2,\;\ldots ,\;n$$

A qualitative analysis of Equation (4) indicates that the relaxation distance $A_{i}$ is inversely proportional to the angular coefficient (slope) of the tangent line approaching the fluid temperature curve at $Z_{i}$. On the other hand, Equation (5) shows a closed-form solution for $A_{i}$.

Consequently, once the fluid temperature profile T(Z) is known, it is easily possible to obtain the tangent line at a region of interest, determine its slope, calculate the relaxation distance $A_{i}$ and, finally, estimate the mass flow rate $M_{i}$ using Equation (2). Therefore, no iterative calculation is necessary. The only remaining difficulty is in determining the characteristics of the term into parentheses in Equation (2). A way to eliminate this problem is further discussed in Section 3.1.4. The linear model simplifies the flow rates calculations and makes available a useful tool for uncertainty estimation as discussed later in Section 3.1.5.

#### 3.1.4. Estimating the Flow Rates from a Fluid Temperature Profile Using the Linear Model

Analyzing the Equation (2), as discussed in Section 3.1.1 and Section 3.1.2, the relaxation distance $A_{i}$ is directly proportional to mass flow rate $M_{i}$ and several properties of the fluid, earth, and wellbore. However, since the inside radius of tubing remains constant along the wellbore and each region is subjected to the same mechanisms of heat transfer, the term in parentheses is independent of depth and can be considered constant for each region. In this case, Equation (2) can be rewritten as:

(6) $$A_{i}=M_{i}\times \left(\frac{c_{p}}{2\pi r_{tub}U_{c}}\right)=M_{i}\times c_{Z},\;i=1,\;2,\;\ldots ,\;n$$

where c_z_ is a constant along the wellbore.

From Figure 3, the mass flow rate $M_{1}$ is equal to the total mass flow rate at surface:

(7) $$M_{1}=M$$

Thus, the mass flowrates $M_{i}$ and $M_{1}$ are related to $A_{i}$ and $A_{1}$ by Equation (8):

(8) $$\frac{A_{i}}{A_{1}}=\frac{M_{i}\times c_{Z}}{M_{1}\times c_{Z}}=\frac{M_{i}}{M_{1}},\;i=1,\;2,\;\ldots ,\;n$$

Consequently, from Equations (7) and (8), the mass flow rates $M_{i}$ are:

(9) $$M_{i}=\frac{A_{i}}{A_{1}}M_{1}=\frac{A_{i}}{A_{1}}M,\;i=1,\;2,\;\ldots ,\;n$$

where $A_{i}$ is given by Equation (5).

Therefore, once the fluid temperature profile along the wellbore, the geothermal temperature, and the total mass flow at surface are all known, a second method to estimate the flow rates in a multiple-zone water-injection well can be used, which is described below:

1. Identify the injection zones and determine the steady-state regions from the temperature profile;
2. Obtain the linear approximation for each steady-state region along the wellbore;
3. Determine the relaxation distances *A*_1_, *A*_2_, …, *A*_n_ from Equation (5);
4. Determine the flow rates *M*_1_, *M*_2_, …, *M*_n_ from Equation (9);
5. Calculate the flow rates *M*_1_′, *M*_2_′, …, *M*_n_′:
  (10) $${M_{i}}^{\prime }=M_{i}-M_{i+1},\;i=1,\;2,\;\ldots ,n$$

#### 3.1.5. Uncertainty Calculation

Previous research [2,7,9] presented methods for estimating the zonal flow rates in a wellbore from temperature surveys. A qualitative discussion about the measurement uncertainty [2] was performed by Muradov and Davies (2012). However, a quantitative analysis of the uncertainties in the flow rates measurement from the fluid temperature measurement along the well was not found in the literature. Therefore, a quantitative uncertainty analysis to understand how errors in the measured temperatures influence the calculated flow rates is presented below.

**Figure 4:**
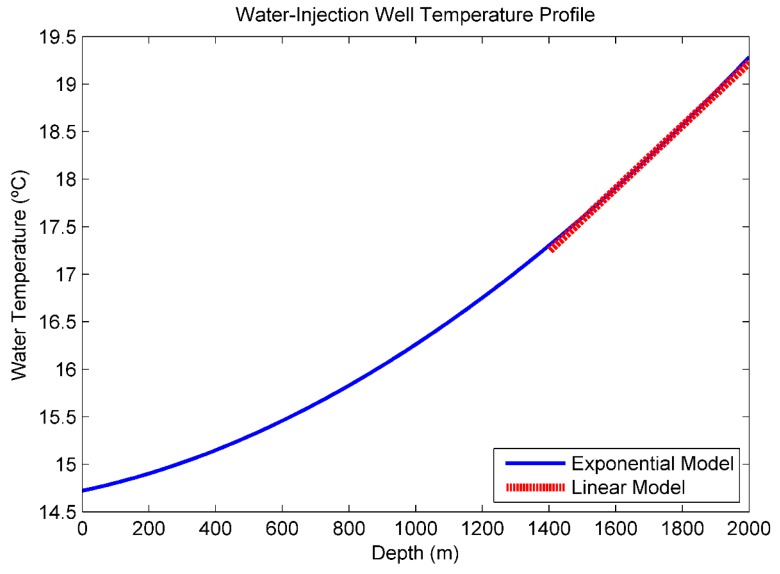
Theoretical Water-Injection Well Fluid Temperature Profile. The blue line represents the exponential Ramey model. The red dots represent the linear approximation. In this example [5], it is assumed to be a single-zone water-injection well with the following characteristics; injection rate: 4790 bbl/d (8.80 m³/s); surface water temperature: 58.5 °F (14.7 °C); no tubing; inside diameter of casing: 6.366 in (0.16 m); geothermal gradient: 0.0083 °F/ft (0.0151 °C/m); geothermal temperature at surface: 70.0 °F (21.11 °C).

In Figure 4, a theoretical example [5] of a fluid temperature profile along a single-zone water-injection well is illustrated. The exponential curve was obtained from Equation (1). The linear approximation was determined using Equation (4) at *Z*_i_ = 1700 m.

The fluid temperature *T*(*Z*) varies exponentially throughout the wellbore. However, as the depth increases the temperature profile becomes nearly linear. Therefore, it is reasonable to suggest a linear approximation of the model proposed by Ramey (1962) for the temperature distribution along the water-injection well [5].

Suppose it is desired to calculate the fluid temperature at *Z* = 1800 m. By the exponential Equation (1), *T* (1800 m) = 65.60 °F (18.67 °C). By the linear Equation (4), *T* (1800 m) = 65.58 °F (18.66 °C). Therefore, at this depth, the Linear model is a very close approximation of the Ramey model, and the systematic error, inherent to the linearization process, is negligible.

From Equation (1), it is difficult to estimate the measurement uncertainty using the Ramey model because the injected flow rates are transcendental functions of fluid temperature. However, since the linearization error is negligible, it is easier to estimate the measurement uncertainty from the Linear model described in Equation (4).

Considering the fluid and geothermal temperatures at each depth along the wellbore can be represented by a measured value and an measurement uncertainty, the temperatures T(Z), $T\left(Z_{i}\right)$, and $T_{geo}\left(Z_{i}\right)$ in Equation (5) can be described by:

(11) $$T\left(Z\right)=\bar{T\left(Z\right)}\pm ∆T\left(Z\right)$$

(12) $$T\left(Z_{i}\right)=\bar{T\left(Z_{i}\right)}\pm ∆T\left(Z_{i}\right)$$

(13) $$T_{geo}\left(Z_{i}\right)=\bar{T_{geo}\left(Z_{i}\right)}\pm ∆T_{geo}\left(Z_{i}\right)$$

where $\bar{T\left(Z\right)}$, $\bar{T\left(Z_{i}\right)}$, and $\bar{T_{geo}\left(Z_{i}\right)}$ are the measured values and ∆T(Z), $∆T\left(Z_{i}\right)$, and $∆T_{geo}\left(Z_{i}\right)$ are the measurement uncertainties.

As described in Appendix B, neglecting the uncertainties in depth measurements and propagating the temperature uncertainties, the random error for the relaxation distance $A_{i}$ is:

(14) $$∆A_{i}=\left[\frac{∆T_{geo}\left(Z_{i}\right)+∆T\left(Z_{i}\right)}{T_{geo}\left(Z_{i}\right)-T\left(Z_{i}\right)}+\frac{∆T\left(Z\right)+∆T\left(Z_{i}\right)}{T\left(Z\right)-T\left(Z_{i}\right)}\right]A_{i},\;i=1,\;2,\;\ldots ,\;n$$

Similarly, the random error for the flow rate $M_{i}$ in each steady-state region is:

(15) $$∆M_{i}=\left[\frac{∆A_{i}}{A_{i}}+\frac{∆A_{1}}{A_{1}}+\frac{∆M}{M}\right]M_{i},\;i=1,\;2,\;\ldots ,\;n$$

where ∆M is the measurement uncertainty for the total flow rate at surface.

Finally, the measurement uncertainties for the injected flow rates are:

(16) $$∆{M_{i}}^{\prime }=∆M_{i}+∆M_{i+1},\;i=1,\;2,\;\ldots ,\;n$$

### 3.2. Modeling a Flow Rate Meter in Multiple-Zone Water-Injection Wells

After consolidating the theoretical aspects, a computer program that calculates the injected flow rates in a multiple-zone water-injection well was implemented. To verify that the linearization error is negligible, both the Ramey and Linear models were used to estimate the water temperature.

First, it runs an iterative process to calculate the geothermal gradient. It starts from the interval defined between 0.01 and 0.03 °C/m. The iterative process is aborted if the error is minor or equal to 0.001 °C/m, or after 10 iterations. Next, it runs another iterative process to calculate the injected flow rates using the Ramey model. It starts from an interval defined by the program user. The iterative process is aborted if the error is minor or equal to 0.001 m³/d, or after 10 iterations. Both algorithms use the Secant Method, a finite difference approximation of the Newton-Rapson Method.

After, it recalculates the injected flow rates using the Linear model and estimates the measurement uncertainties. Finally, it generates the fluid temperature profile and plots a graph comparing the theoretical Ramey and Linear models with the experimental profile obtained in the field.

### 3.3. Data Acquisition

In the experimental part, the fluid temperature profile from a dual-zone water-injection well located in the Northeast Brazilian region was collected. A temperature sensor was introduced and moved through the tubing, logging the fluid temperature approximately every 50 m along the wellbore. In each point, the temperature sensor performed one measurement per second, with a resolution of 0.1 °C, for the duration of 5 to 10 min, thus totaling 300 to 600 temperature measurements. Finally, a flow sensor was also introduced through the well, measuring the flow rates in each injection zone. Another flow sensor measured the total flow at the surface.

### 3.4. Data Processing and Analysis

First, the mean value and the standard deviation of the mean from the 300 to 600 measurements performed at each depth along the wellbore were calculated, thereby building the processed temperature profile. Next, the processed temperature profile was qualitatively analyzed in order to identify the injection zones and determine the steady-state regions. After, the flow rates in each injection zone were calculated by the computer program from both Ramey and Linear models. Finally, the calculated and measured flow rates were compared.

## 4. Results and Discussion

**Figure 5:**
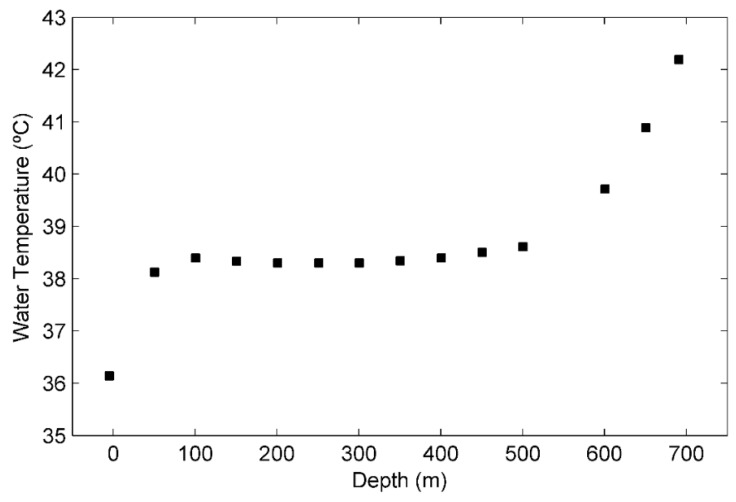
Fluid temperature profile built after processing the field data.

**Figure 6:**
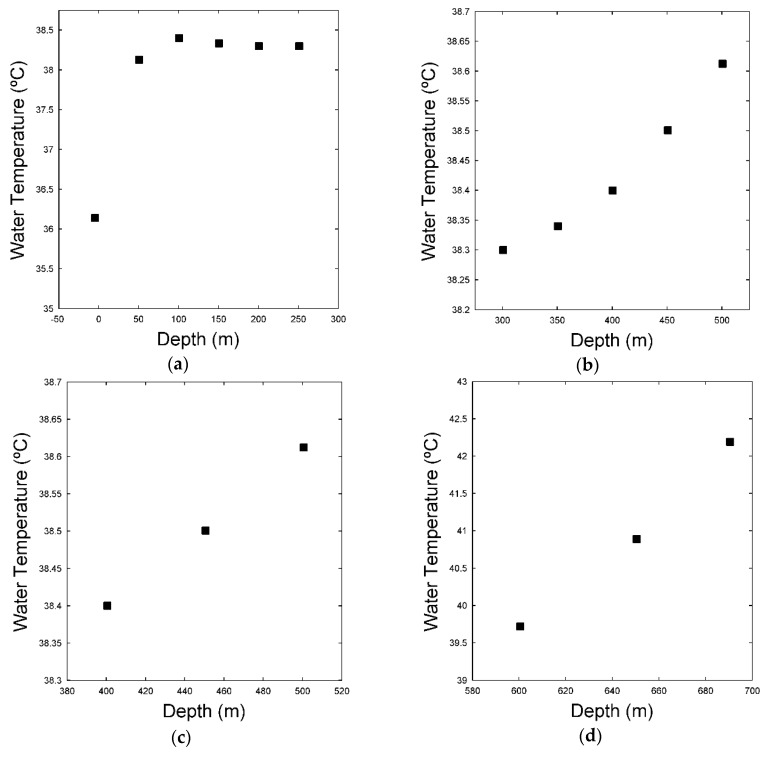
Fluid temperature profile. (**a**) The graph was stretched out from 0 m to 250 m; (**b**) The graph was stretched out from 300 m to 500 m; (**c**) The graph was stretched out from 400 m to 500 m; (**d**) The graph was stretched out from 600 m to 690 m.

The field data collected from a dual-zone water-injection well were processed as described in Section 3.4. The results are shown in Table 1. The mean fluid temperature and the standard deviation of the mean were respectively considered the measured value and the measurement uncertainty at each depth along the wellbore. The experimental fluid temperature profile is illustrated in Figure 5. From the well schematics, it was known that the water-injection well had two injection zones at 538 m and 695 m. The changes in the water temperature gradient along the wellbore from 100 m to 500 m are not clearly observable in Figure 5 because there is an abrupt transition in temperature near the bottomhole (from 600 m to 700 m). Therefore, to identify the injection zones, determine the steady-state regions, and perceive the changes in water temperature gradient, it is necessary to stretch out the graph as viewed in Figure 6.

**Figure 7:**
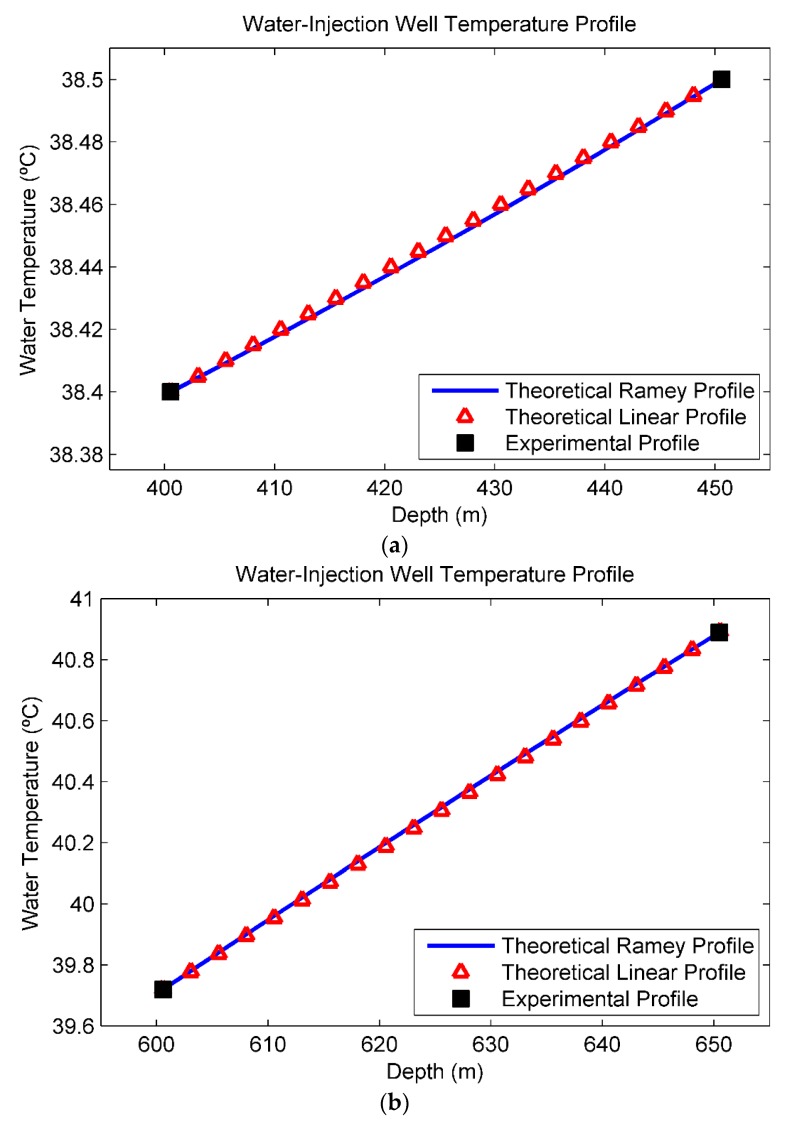
Theoretical (Ramey and Linear) and experimental temperature profiles in the steady-state regions. (**a**) Region upstream from the first injection zone (538 m). The fluid temperature gradient is about 0.002 °C/m; (**b**) Region upstream from the second injection zone (695 m). The fluid temperature gradient is about 0.02 °C/m.

Analyzing Figure 6a, in discarding the temperature measurement taken at surface, there is practically no heat transmission and the fluid temperature remains approximately constant until 250 m. It occurs because at this depth the annular space between the injection and casing strings is filled with air, which is a thermal insulator. From 300 m to 500 m (Figure 6b), the temperature starts to increase in an apparently exponential behavior. From 400 m to 500 m (Figure 6c), immediately before the first injection zone (538 m), the temperature profile has a clearly linear course. Finally, from 600 m to 690 m (Figure 6d), immediately before the second injection zone (695 m), the temperature profile also displays linear behavior. To apply the Ramey model and, consequently, the Linear model, it is necessary to consider a range of depths in steady-state. In this sense, the measurements taken near the surface were discarded. Moreover, because the temperature measurements at 500 m and 690 m were taken at depths too close to the injection zones (538 m and 695 m), the steady-state condition is also not satisfied. Therefore, these two points were also discarded and the ranges from 400 m to 450 m and from 600 m to 650 m were considered as the steady-state regions. The theoretical (Ramey and Linear) and experimental temperature profiles in the steady-state regions are plotted in Figure 7.

In Figure 7a the steady-state region upstream from the first injection zone (538 m) is illustrated. The linear approximation was performed by determining the line passing through the measured points at 400 m and 450 m. Figure 7b shows the steady-state region upstream from the second injection zone (695 m). The linear approximation was performed by determining the line passing through the measured points at 600 m and 650 m. The fluid temperature gradients are about 0.002 °C/m and 0.02 °C/m. In both regions of interest, the exponential and linear curves are overlapping. Therefore, in these ranges, the linearization error is negligible.

The theoretically calculated and experimentally measured injected flow rates are shown in Table 2. The uncertainties for the measured flow rates are unknown and were not represented. The absolute uncertainties for the calculated flow rates from the Ramey and Linear models are about 1.20 m³/d and 1.35 m³/d, respectively. For the first injection zone, this is equivalent to 1.22% and 1.40% of the calculated values from the Ramey and Linear models, respectively. For the second injection zone, these absolute uncertainties are equivalent to 10.47% and 9.88% of the calculated values from the Ramey and Linear models, respectively. Therefore, the relative uncertainty increases as the flow rate decreases.

Moreover, the calculated flow rates from the Ramey and Linear models presented a difference of only 4.58 m³/d and 2.38 m³/d, respectively, to the measured flow rates. Therefore, the two methods presented a satisfactorily accurate measurement. Finally, it is interesting to observe that, in this case, the calculated flow rates from the Linear model were closer to the measured flow rates than the calculated flow rates from the Ramey model. A reasonable interpretation of this issue is that, in the Ramey model, unlike the Linear model, it is necessary to know the thermal properties of fluid and earth; the injection time; and the constructive characteristics of the well (dimensions and materials of tubing, casing, and cement). Therefore, to calculate the injected flow rates from Ramey model it is necessary to suppose these properties, leading to increases in the measurement error.

## 5. Conclusions

This work is a contribution to the development of flow sensors for the oil and gas industry. It presented two methods to measure the flow rates into multiple-zone water-injection wells from fluid temperature profiles. First, a method using the Ramey model, which defines the fluid temperature as a transcendental, exponential function of flow, was described. In this method, the downhole flow rates have to be iteratively calculated. Moreover, several thermal properties and constructive characteristics of the well need to be known to perform the flow rate calculations.

Next, a second method using a Linear model that defines a closed-form solution for the injected flow rates as a function of the fluid temperature was discussed. The obtained results showed that, in the regions of interest, the exponential and linear curves overlap, as illustrated in Figure 7. Therefore, under certain conditions, the linearization error is negligible for practical purposes. The possibility of using a more complex polynomial model to further improve the results is the subject of another work currently in development. The basic idea is to expand the Ramey equation in Taylor Series and rearrange the resultant equation in order to consider a quadratic or cubic approximation.

Besides simplifying the downhole flow rates calculations, the second method made available a useful tool for uncertainty estimation. Thus, an uncertainty analysis was performed. In this approach, the systematic error, inherent to the linearization of the Ramey model, was not taken into account. The random error was estimated from the propagation of uncertainties for the measured temperatures at each depth along the wellbore.

A computer program modeling a flow rate meter in multiple-zone water-injection wells was implemented. It calculates the injected flow rates using both the Ramey (Exponential) and the Linear models, estimates the measurement uncertainties, and compares the theoretical Ramey (Exponential) and Linear models with the experimental profile obtained in the field.

In the experimental part, a fluid temperature profile from a dual-zone water-injection well located in the Northeast Brazilian region was collected. The total flow rate was 110.00 m³/d. The injected flow rates were 93.96 m³/d and 16.04 m³/d at zones 1 and 2, respectively. It was observed that the calculated flow rates from both the Ramey and Linear models presented a satisfactorily accurate measurement. The calculated values were very close to the measured flow rates, presenting a difference of only 4.58 m³/d and 2.38 m³/d, respectively. It was also observed that the relative uncertainty increases as the flow rate decreases. For the first injection zone, the estimated uncertainties were equal to 1.22% and 1.40% of the calculated flow rates from the Ramey and Linear models, respectively. For the second injection zone, the uncertainties were 10.47% and 9.88% of the calculated values from the Ramey and Linear models, respectively. Therefore, both methods were successfully validated and all objectives of this work were achieved.

This approach to measure the downhole flow rates has a limitation due to the temperature sensor and the field procedure used to collect the fluid temperature profile. In the experimental part, the temperature sensor had a resolution of 0.1 °C and the temperature measurements were taken every 50 m. Each steady-state region also had a length of 50 m. Moreover, the measured fluid temperature gradients were 0.002 °C/m and 0.02 °C/m for the steady-state regions 1 and 2, respectively. Thus, the temperature variations in the steady-state regions 1 and 2 were about 0.1 °C and 1 °C, respectively. Consequently, the sensor that was used was able to detect the temperature changes and the obtained results were satisfactory. However, if the first steady-state region had a length of 40 m, for example, the temperature change would be only 0.08 °C and the sensor would not be able to detect this variation. This issue is the subject of another paper currently being prepared.

This limitation can be satisfactorily reduced as better temperature sensors are applied. For example, a typical DTS optic fiber has made downhole temperature measurements with a resolution of 0.01 °C and intervals of 1 m potentially available. Moreover, once the optic fiber is permanently inserted within the injection column, it makes it possible to have an in-line acquisition of the fluid temperature profile along the multiple-zone water-injection well at shorter intervals of time, or even in real-time, thereby offering information in sufficient frequency for the reservoir engineering team.

## Figures and Tables

**Table 1:** Processed field data for a dual-zone water-injection well.

Depth (m)	Number of Measurements	Mean Fluid Temperature (°C)	Standard Deviation of the Mean (°C)
−4.70	358	36.14	0.05
50.60	593	38.127	0.009
100.61	311	38.400	0.003
150.55	310	38.334	0.003
200.74	308	38.300	0.003
251.13	311	38.301	0.001
300.62	306	38.300	0.003
350.55	320	38.340	0.003
400.56	306	38.400	0.003
450.61	322	38.5006	0.0008
500.55	318	38.612	0.002
600.57	318	39.72	0.02
650.50	311	40.89	0.02
690.55	632	42.19	0.01

**Table 2:** Measured and calculated flow rates in m³/d.

Injection Zone	Measured Flow Rate (m³/d)	Calculated Flow Rate from Ramey Model (m³/d)	Calculated Flow Rate from Linear Model (m³/d)
1	93.96	98.54 ± 1.20	96.34 ± 1.35
2	16.04	11.46 ± 1.20	13.66 ± 1.35
Total	110.00	110.00	110.00

## Appendix A

As described in Section 3.1.1, the relaxation distance $A_{i}$ depends on the overall heat-transfer coefficient $U_{c}$. Because of the high thermal conductivity and relatively small radial distance between the flowing fluids and the borehole wall, heat transfer in this region normally can be considered to be steady state [13]. Considering the longitudinal section previously illustrated in Figure 3, the steady-state heat transfer between the flowing fluid and the formation (surrounding rock) is primarily a result of: convection within the tubing; conduction throughout the tubing wall; convection within the fluid-filled annulus; conduction throughout the casing and cement walls; and conduction throughout the formation [13]. Therefore, the overall heat-transfer coefficient $U_{c}$ [13] is given by:

(A1) $$U_{c}=\frac{1}{r_{to}\times \left[\frac{1}{r_{ti}h_{f}}+\frac{ln\left(\frac{r_{to}}{r_{ti}}\right)}{k_{t}}+\frac{1}{r_{ci}h_{an}}+\frac{ln\left(\frac{r_{co}}{r_{ci}}\right)}{k_{c}}+\frac{ln\left(\frac{r_{w}}{r_{co}}\right)}{k_{cem}}+\frac{f\left(t\right)}{k_{e}}\right]}$$

where $r_{to}$ is the outside radius of the tubing, $r_{ti}$ is the inside radius of the tubing, $h_{f}$ is the local film coefficient of heat transfer for fluid inside tubing, $k_{t}$ is the thermal conductivity of the tubing, $r_{ci}$ is the inside radius of the casing, $r_{co}$ is the outside radius of the casing, $h_{an}$ is the local film coefficient of heat transfer for fluid in annulus, $k_{c}$ is the thermal conductivity of the casing, $r_{w}$ is the outside radius of the wellbore; $k_{cem}$ is the thermal conductivity of the cement, and f(t) is the transient heat-conduction time function for earth.

The local film coefficient of heat transfer for fluid inside tubing is equal to:

(A2) $$h_{f}=\frac{Nnu_{f}\times k_{t}}{2\times r_{ti}}$$

where $Nnu_{f}$ is the Nusselt number inside tubing.

In turn, the local film coefficient of heat transfer for fluid in annulus is equal to:

(A3) $$h_{an}=\frac{Nnu_{an}\times k_{c}}{2\times r_{ci}}$$

where $Nnu_{an}$ is the Nusselt number in annulus.

For Reynolds numbers above 10^4^, the Nusselt numbers are related to Reynolds and Prandtl numbers [13] by:

(A4) $$Nnu_{f}=0.023\times Nre_{f}^{0.8}\times Npr_{f}^{0.3}$$

and

(A5) $$Nnu_{an}=0.023\times Nre_{an}^{0.8}\times Npr_{an}^{0.3}$$

The Prandtl numbers in tubing and annulus are given by:

(A6) $$Npr_{f}=\frac{c_{f}\times \mu_{f}}{k_{t}}$$

and

(A7) $$Npr_{an}=\frac{c_{an}\times \mu_{an}}{k_{c}}$$

where $c_{f}$ is the specific heat of fluid inside tubing, $\mu_{f}$ is the viscosity of fluid inside tubing,$\;c_{an}$ is the specific heat of fluid in annulus and $\mu_{an}$ is the viscosity of fluid in annulus.

The Reynold numbers in tubing and annulus are given by:

(A8) $$Nre_{f}=\frac{2r_{ti}M_{f}}{\mu_{f}\pi {r_{ti}}^{2}}$$

and

(A9) $$Nre_{an}=\frac{2r_{ci}M_{an}}{\mu_{an}\pi {r_{ci}}^{2}}$$

where $M_{f}$ is the mass flow rate of fluid inside tubing and $M_{an}$ is the mass flow rate of fluid in annulus.

Finally, $t_{Dw}$ is defined as:

(A10) $$t_{Dw}=\frac{\alpha t}{{r_{w}}^{2}}$$

where α is the thermal diffusivity of earth and t is the time of injection. The transient heat-conduction time function for earth f(t) [13] is:

(A11) $$f\left(t\right)=1.1281\sqrt{t_{Dw}}\left(1-0.3\sqrt{t_{Dw}}\right),\;if\;t_{Dw}\leq 1.5$$

or

(A12) $$f\left(t\right)=\left[0.4063+0.5ln\left(t_{Dw}\right)\right]\left(1+\frac{0.6}{t_{Dw}}\right),\;if\;t_{Dw}>1.5$$

Therefore, as discussed in Section 3.1.1, the overall heat-transfer coefficient $U_{c}$ depends on the thermal properties of fluid and earth; the injection time; and the constructive characteristics of the well (dimensions and materials of tubing, casing, and cement). Oftentimes, some of these characteristics are not easily known and it is necessary to suppose them to calculate the mass flow rates in each steady-state region using the Ramey model. Furthermore, because the Ramey model for fluid temperature is a transcendental, exponential function of the flow rate, the calculations have to be iteratively performed, increasing the difficulty to apply the first method to estimate the downhole zonal flow rates, as described in Section 3.1.2. However, using the Linear model described in Section 3.1.3, all these properties and constructive characteristics may be ignored and the calculations may be directly performed as the method discussed in Section 3.1.4.

## Appendix B

### B.1. Linearization of the Ramey Model

Considering the heat transfer in a single-zone water-injection well is steady state, the Ramey model [5] for fluid temperature as a function of depth is given by:

(B1) $$T\left(Z\right)=aZ+b-aA+\left(T_{0}+aA-b\right)e^{-\frac{Z}{A}}$$

where a is the geothermal gradient, b is the geothermal temperature at surface, A is the relaxation distance, and $T_{0}$ is the fluid temperature at surface.

The exponential term may be expanded in Taylor series at Z = 0 considering a linear approximation:

(B2) $$e^{-\frac{Z}{A}}\approx {\left[e^{-\frac{Z}{A}}\right]}_{z=0}{\left(Z-0\right)}^{0}+{\left[\frac{d}{dZ}\left(e^{-\frac{Z}{A}}\right)\right]}_{Z=0}\frac{{\left(Z-0\right)}^{1}}{1!}$$

where

(B3) $${\left[e^{-\frac{Z}{A}}\right]}_{Z=0}=1$$

and

(B4) $${\left[\frac{d}{dZ}\left(e^{-\frac{Z}{A}}\right)\right]}_{Z=0}={\left[-\frac{1}{A}e^{-\frac{Z}{A}}\right]}_{Z=0}=-\frac{1}{A}$$

Therefore, the Equation (B1) can be rewritten as:

(B5) $$T\left(Z\right)\approx aZ+b-aA+\left(T_{0}+aA-b\right)\left[1-\frac{1}{A}Z\right]\approx T_{0}+\left(\frac{b-T_{0}}{A}\right)Z$$

Analogously, for a multiple-zone water-injection well, the fluid temperature in each steady-state region can be approximated at $Z_{i}$ by:

(B6) $$T\left(Z\right)\approx T\left(Z_{i}\right)+\left[\frac{T_{geo}\left(Z_{i}\right)-T\left(Z_{i}\right)}{A_{i}}\right]\left(Z-Z_{i}\right),\;i=1,\;2,\;\ldots ,\;n$$

where $T\left(Z_{i}\right)$ is the fluid temperature at $Z_{i}$; $T_{geo}\left(Z_{i}\right)$ is the geothermal temperature at $Z_{i}$; and $A_{i}$ is the relaxation distance in each region.

### B.2. Uncertainty Calculations from the Linear Model

Solving the Equation (B6) for $A_{i}$:

(B7) $$A_{i}=\left[\frac{T_{geo}\left(Z_{i}\right)-T\left(Z_{i}\right)}{T\left(Z\right)-T\left(Z_{i}\right)}\right]\times \left(Z-Z_{i}\right),\;i=1,\;2,\;\ldots ,\;n$$

Neglecting the depth uncertainties, from the propagation of errors approach the uncertainty in the relaxation distance $A_{i}$ is given by:

(B8) $$\frac{∆A_{i}}{A_{i}}=\frac{∆\left[T_{geo}\left(Z_{i}\right)-T\left(Z_{i}\right)\right]}{T_{geo}\left(Z_{i}\right)-T\left(Z_{i}\right)}+\frac{∆\left[T\left(Z\right)-T\left(Z_{i}\right)\right]}{T\left(Z\right)-T\left(Z_{i}\right)},\;i=1,\;2,\;\ldots ,\;n$$

Considering the uncertainties in the measured temperatures:

(B9) $$∆A_{i}=\left[\frac{∆T_{geo}\left(Z_{i}\right)+∆T\left(Z_{i}\right)}{T_{geo}\left(Z_{i}\right)-T\left(Z_{i}\right)}+\frac{∆T\left(Z\right)+∆T\left(Z_{i}\right)}{T\left(Z\right)-T\left(Z_{i}\right)}\right]A_{i},\;i=1,\;2,\;\ldots ,\;n$$

As discussed in Section 3.1.4, the mass flow rates $M_{i}$ in the steady-state regions are equal to:

(B10) $$M_{i}=\frac{A_{i}}{A_{1}}M_{1}=\frac{A_{i}}{A_{1}}M,\;i=1,\;2,\;\ldots ,\;n$$

Considering the uncertainties in the relaxation distances and in the total flow rate:

(B11) $$∆M_{i}=\left[\frac{∆A_{i}}{A_{i}}+\frac{∆A_{1}}{A_{1}}+\frac{∆M}{M}\right]M_{i},\;i=1,\;2,\;\ldots ,\;n$$

Finally, the injected flow rates are given by:

(B12) $${M_{i}}^{\prime }=M_{i}-M_{i+1},\;i=1,\;2,\;\ldots ,\;n$$

Therefore, the uncertainties in the injected flow rates are equal to:

(B13) $$∆{M_{i}}^{\prime }=∆M_{i}+∆M_{i+1},\;i=1,\;2,\;\ldots ,\;n$$
